# Ilizarov technique in the treatment of bone defects of the radius and ulna: a systematic review and meta-analysis

**DOI:** 10.1186/s13018-023-04126-4

**Published:** 2023-08-30

**Authors:** Yimurang Hamiti, Ainizier Yalikun, Cheng Lu, Aihemaitijiang Yusufu, Maimaiaili Yushan

**Affiliations:** 1 Department of Microrepair and Reconstructive Surgery, The First Affiliated Hospital of Xinjiang Medical University, Ürümqi, Xinjiang People’s Republic of China; 2 Department of Orthopedic Surgery, The Fourth Affiliated Hospital of Xinjiang Medical University, Traditional Chinese Medicine Hospital of Xinjiang Uyghur Autonomous Region, Ürümqi, Xinjiang People’s Republic of China

**Keywords:** Ilizarov technique, Distraction osteogenesis, Bone defect, Radius, Ulna

## Abstract

**Purpose:**

The objective of this systematic review and meta-analysis was to assess the efficacy of the Ilizarov method in the treatment of radius and ulna bone defects.

**Methods:**

The PubMed, Embase, Web of Science, Cochrane Library, Ovid MEDLINE, and Scopus databases were searched for articles published up to May 2023. The quality of the studies was evaluated using a modified version of the Newcastle–Ottawa scale. The effect size and confidence intervals at 95% for the main results were calculated. The heterogeneity was evaluated. The demographic data, defect size (DS), external fixation time (EFT), external fixation index (EFI), and complications were extracted and analyzed using the Stata version 16.

**Results:**

This meta-analysis identified and included seven studies involving 98 patients. The union rate of 100% was reported in all studies. According to the findings of the single-arm meta-analysis, the pooled DS was 3.42 cm (95% CI [2.64, 4.21], *I*^*2*^ = 53.5%, *P* = 0.045), EFT was 148.43 days (95% CI [97.49, 199.38], *I*^*2*^ = 91.9%, *P* = 0.000), and EFI was 41.32 days/cm (95% CI [35.72, 46.91], *I*^*2*^ = 62.2%, *P* = 0.021). Pin tract infection was the most common complication, as reported in six studies.

**Conclusion:**

The findings of the present meta-analysis indicate that the Ilizarov technique is a successful treatment option for bone defects in the radius and ulna. This method has demonstrated efficacy in achieving expected clinical outcomes.

**Supplementary Information:**

The online version contains supplementary material available at 10.1186/s13018-023-04126-4.

## Introduction

Forearm bone defects may result from various causes, including high-energy trauma, excision of contaminated and devascularized bone fragments in open fractures, resection of bone tumors such as hereditary multiple exostosis, or extensive debridement of infected nonunion [1–14]. The effective management of the conditions mentioned above poses a significant challenge in restoring the biomechanics of the elbow and wrist. This is primarily due to the critical proximity of essential neurovascular structures and the imperative need to preserve the supination, pronation, and range of motion of the adjacent joints. Consequently, orthopedic surgeons have encountered persistent difficulties in this regard [8, 14].

Literature on this topic reveals that despite the use of various techniques, the outcomes are not identical and that there is still debate over which technique to employ. The Ilizarov method, which is based on distraction osteogenesis, is currently being utilized successfully in the upper extremities [8–15]. A systematic review and meta-analysis conducted previously have demonstrated that distraction osteogenesis provides specific benefits in the field of hand surgery [15]. Despite the existence of several case series on distraction osteogenesis in the forearm within the literature, there is a scarcity of systematic research to provide guidance for surgeons. A definitive agreement regarding the effectiveness and limitations of this procedure has yet to be established.

The aim of this systematic review was to review the extant research studies pertaining to the utilization of Ilizarov technique for the management of bone defects in the radius and ulna, and to conduct a meta-analysis to assess the effectiveness of these methods. To the best of our knowledge, this is the first systematic review and meta-analysis in this respect.

## Materials and methods

### Search strategy

The present meta-analysis strictly followed the principles of the Preferred Reporting Items for Systematic Reviews and Meta-Analyses (PRISMA) statement. It was prospectively registered in the PROSPERO registry (CRD42023436766). Three reviewers searched the following databases up to May 2023 independently for the identifications of studies: PubMed, Embase, Web of Science, Cochrane Library, Ovid MEDLINE, and Scopus databases. The retrieval strategy was mainly composed of MeSH subject words and free words. The search strategy encompassed the utilization of primary search terms, including Ilizarov technique, distraction osteogenesis, bone loss, bone defect, and upper extremity. Detailed search terms are provided in Additional file 1. The evaluation of the quality of the studies was conducted through a modified version of the Newcastle–Ottawa scale, which was applied to the studies that were included in the analysis [16].

### Selection criteria

The following eligibility criteria were performed in the selection of the articles: (1) population: patients with bone defects of the radius and ulna; (2) intervention: Ilizarov method based on distraction osteogenesis; (3) outcomes: defect size, EFT, EFI, and complications. The eligible study included two above-mentioned outcomes at least; (4) article types: any type of the articles, excluding case reports and reviews. The exclusion criteria were as follows: (1) summaries, conference abstracts, reviews, and other non-authoritative researches; (2) studies with incomplete data; (3) repeated publications or studies that used the same data for multiple articles.

### Data extraction

Two authors independently extracted all eligible data that met the established criteria. Discrepancies were resolved by discussion with each other. The following data were extracted from each included study: first author, publication year, study design, number of patients, mean age, gender, site, mean previous surgical procedures, mean follow-up duration, DS, bone union, EFT, EFI, and complications per patient, complications (pin track infection, axial deviation, bone grafting, soft tissue incarceration, delayed union or nonunion, joint stiffness, refracture, dislocation of the radial head, pin loosening, and pain).

### Statistical analysis

All relevant results were presented in the form of proportions (e.g., bone union) or quantitative data (e.g., defect size, EFT, EFI). Summarized estimates of effect size have been established for each relevant outcome. The present analysis conducted a single-arm meta-analysis to examine DS, EFT, and EFI, with their corresponding 95% confidence intervals. The mean for DS and EFT was used to perform subgroup analysis. It is notable that all studies included in this analysis were retrospective case series. Statistical heterogeneity among studies was assessed by inspecting the study-specific magnitudes of effect and the heterogeneity (*I*^*2*^) statistics. If *P* < 0.05 or *I*^*2*^ > 50%, heterogeneity was recognized as significant, and the random-effects model calculated the pooled data estimate. Otherwise, the fixed-effects model was used. In order to evaluate the possible sources of heterogeneity, sensitivity analyses were conducted. Assessment of publication bias was evaluated by Begg's funnel plot. The statistical analysis was conducted with Stata 16 (STATA, College Station, USA).

## Results

### Included literature

**Fig. 1:**
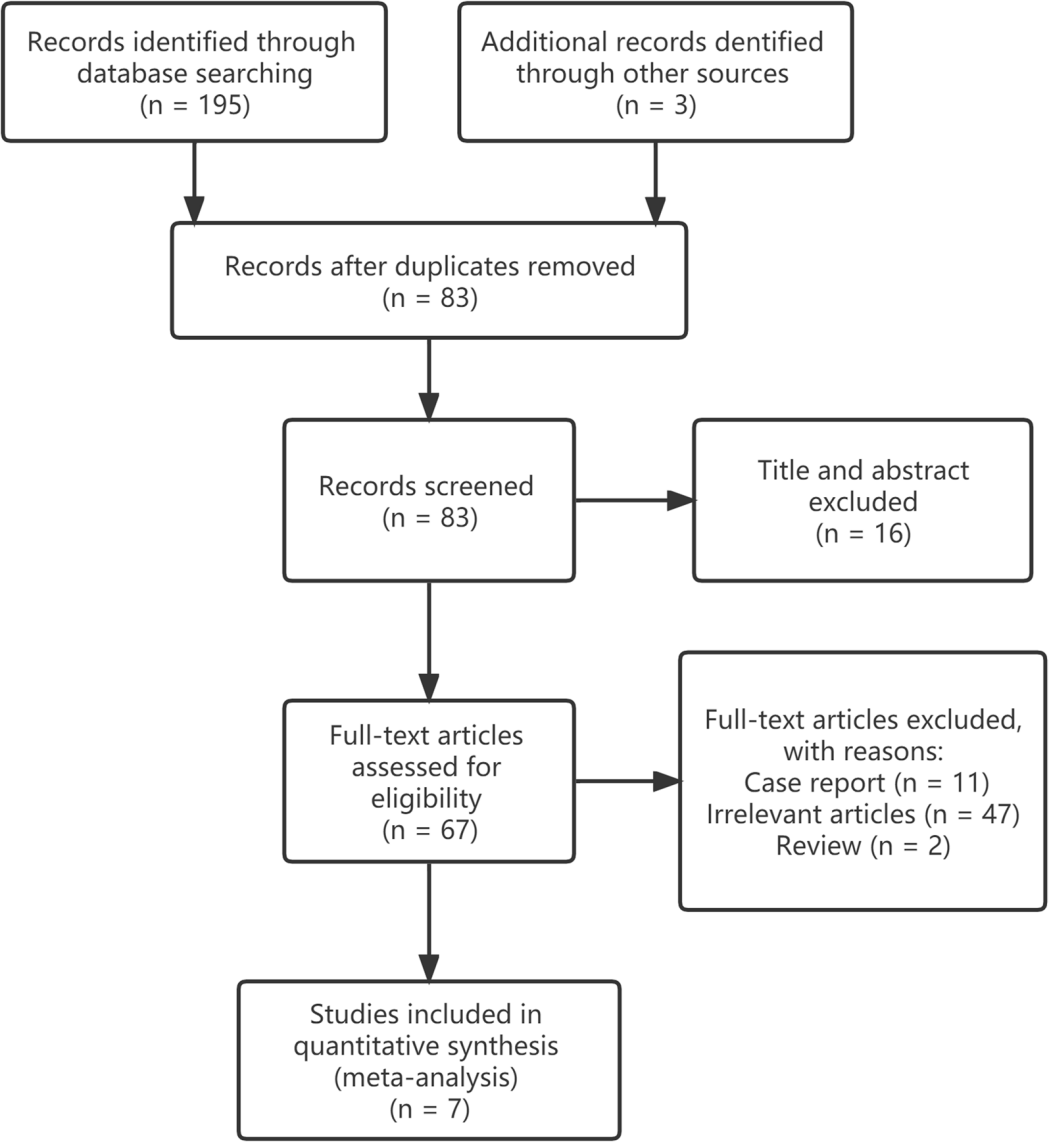
Inclusion flowchart

Regarding the characteristics of the study, a visual representation in the form of a flow diagram was utilized to illustrate the search methodology, and the outcome was succinctly presented in Fig. 1. A total of 198 articles were searched; after eliminating duplicates and screening the title and abstract, 131 were excluded. Then, 60 articles were excluded from the 83 studies according to the inclusion criteria. Ultimately, seven studies met the inclusion and exclusion criteria in the systematic review by reviewing the full-text articles [9–15]. All of the seven studies were retrospective case series. Table 1 indicates the NOS-based quality evaluation of the retrospective studies that were included in the analysis. The median score of NOS was five. Studies with five positive answers were defined as good quality.

**Table 1:** Assessment of the included studies according to the modified Newcastle–Ottawa scale

Parameter	Liu [8]	Zhu [9]	Ahmed [10]	Mader [11]	Zhang [12]	Liu [13]	Smith [14]
Study population clearly defined	Yes	Yes	Yes	Yes	Yes	Yes	Yes
Consecutive patients included	Yes	Yes	Yes	Yes	Yes	Yes	Yes
Selection of controls	No	No	No	No	No	No	No
Definition of controls	No	No	No	No	No	No	No
Assessment of outcome well defined	Yes	Yes	Yes	Yes	Yes	Yes	Yes
Complications well defined	Yes	Yes	Yes	Yes	Yes	Yes	Yes
Follow-up long enough for outcomes to occur (> 1.5 years)	Yes	Yes	Yes	Yes	Yes	Yes	Yes

Studies with five positive answers were defined as good quality

### Patient information

The studies were published between 2003 and 2021. A total of 98 patients with bone defects of the radius and ulna treated by the Ilizarov technique were included in our study. The mean age of all patients was 27.4 years (range, 2–62 years). Patients had an average of 1.7 (range, 0–8) previous surgical procedures before receiving the treatment. The mean length of follow-up was 36.3 months (range, 21–192 months) in the patients. Table 2 summarizes the baseline characteristics of the included studies and patients.

**Table 2:** Characteristics of studies included in the meta-analysis

Study	Study design	Number of patients (*n*)	Gender (male/female)	Mean age (years)	Site (Radius/Ulna)	Mean follow-up (months)	Mean previous operations (per patient)
Liu [8]	RS	12	10/2	39.0 (23–57)	10/2	28.2 (24–36)	2.2 (0–4)
Zhu [9]	RS	19	12/7	37.4 (–62)	11/8	35.4 (24–55)	2.7 (1 –5)
Ahmed [10]	RS	12	8/4	8.7 (7.–10)	0/12	33.2 (24–48)	0
Mader [11]	RS	7	4/3	9.5 (2–16)	2/8	33.0 (22–90)	0
Zhang [12]	RS	16	10/6	38.3 (19–62)	9/7	39.6 (26–55)	2.38 (1–5)
Liu [13]	RS	21	12/9	27.1 (15–56)	8/13	77.5 (21–136)	3.2 (1–8)
Smith [14]	RS	11	7/4	32.0 (3–50)	nr	74.4 (27.6–192)	1.4

*RS* Retrospective case series, *nr* Not reported

### Outcomes

**Fig. 2:**
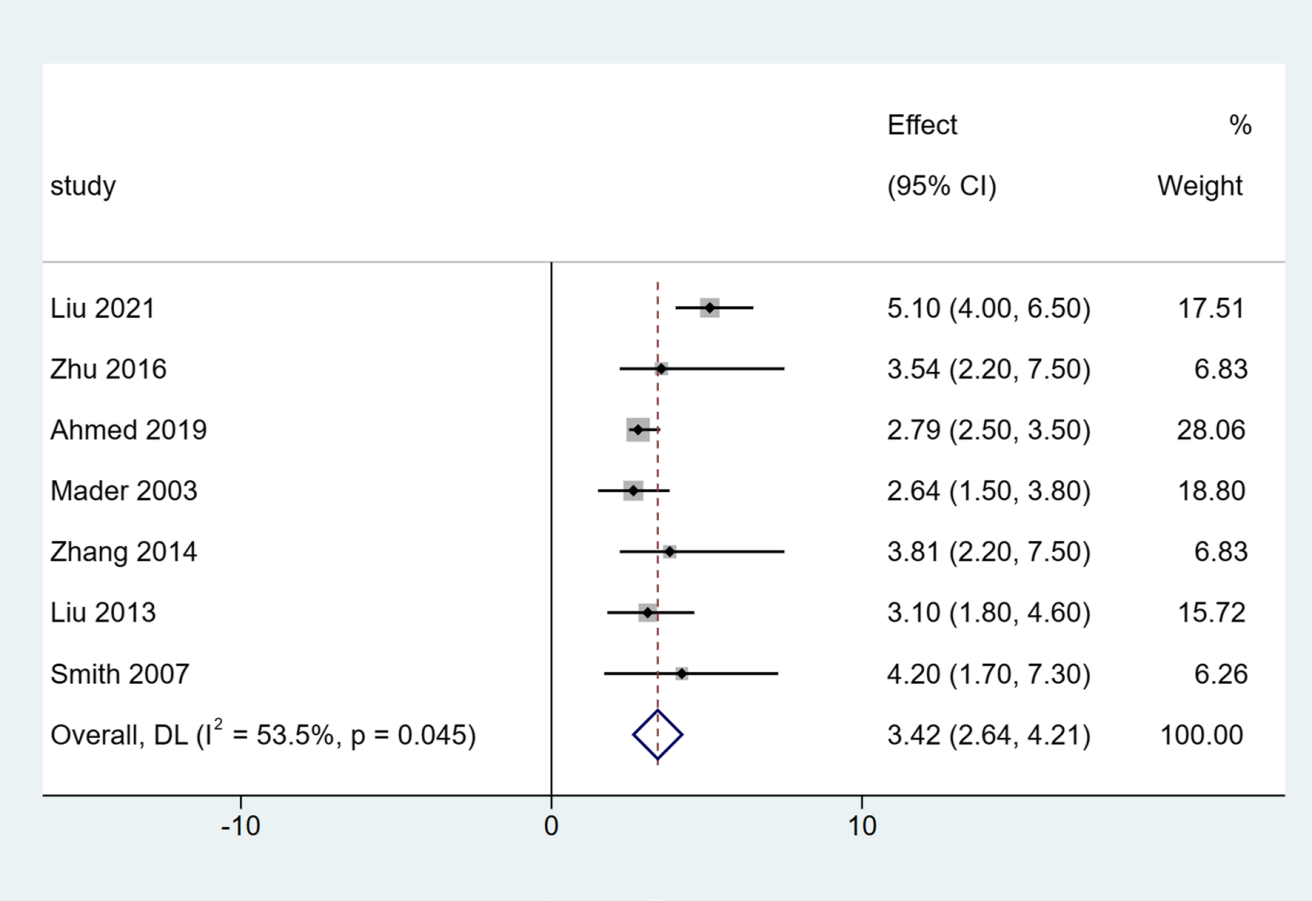
Forest plot of summarized estimates for DS

**Fig. 3:**
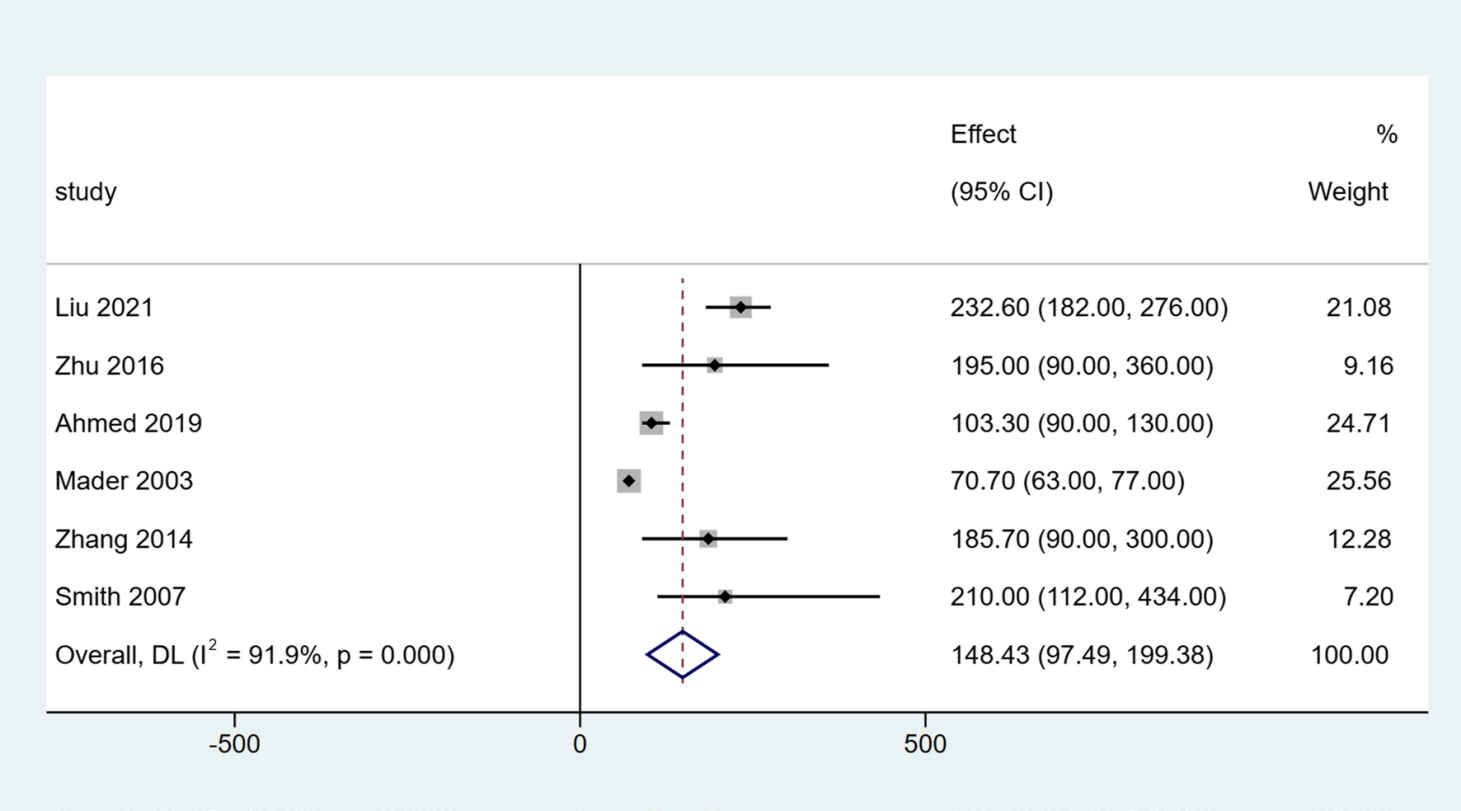
Forest plot of summarized estimates for EFT

**Fig. 4:**
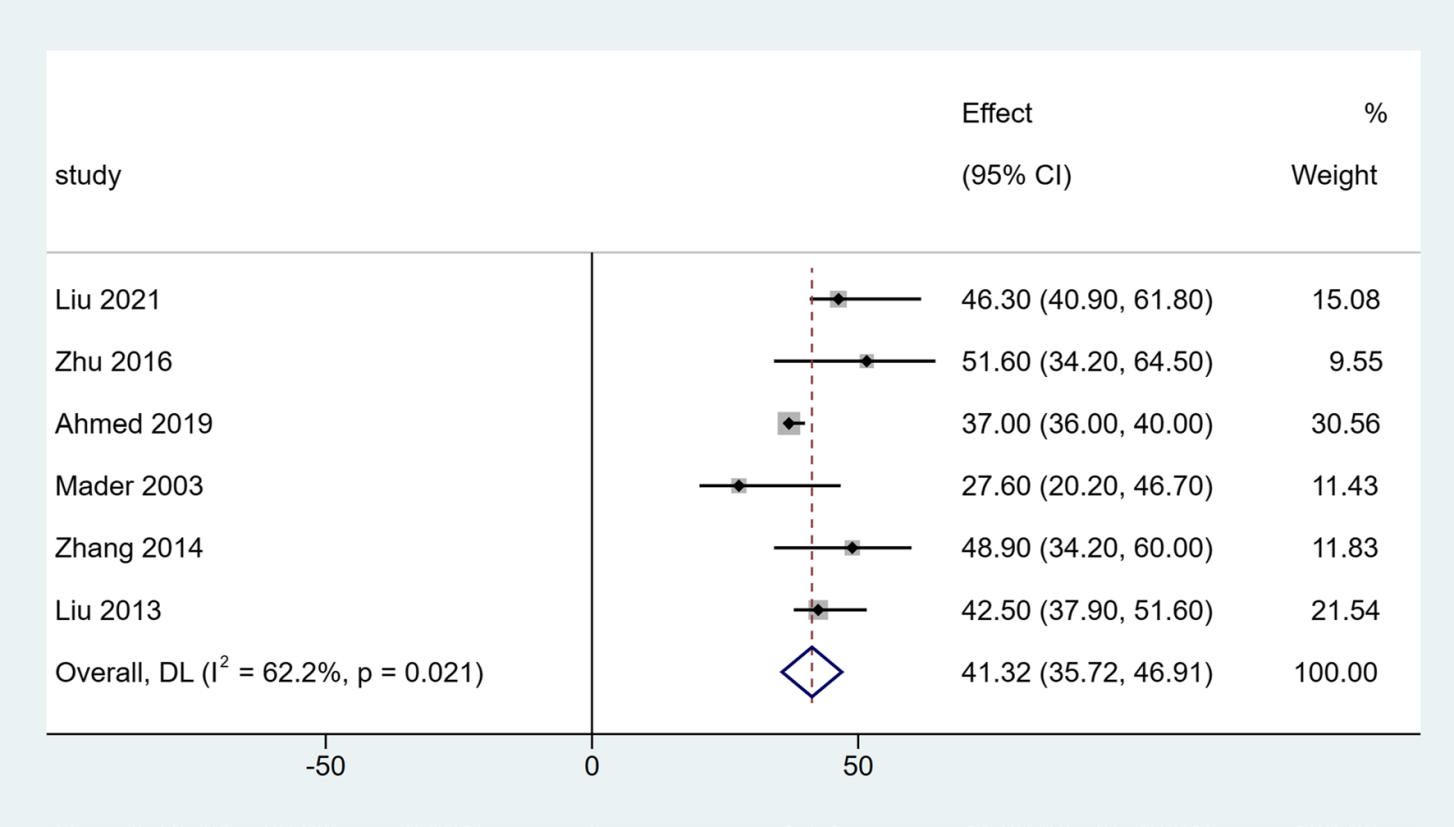
Forest plot of summarized estimates for EFI

The clinical outcomes of the included studies are summarized in Table 3. All studies included in the analysis reported DS in all patients. After a single-arm meta-analysis, the pooled median DS was 3.42 cm (95% CI [2.64, 4.21], *I*^*2*^ = 53.5%, *P* = 0.045), as shown in Fig. 2. There were six studies that reported the EFT and EFI. After summarized estimates, the pooled median EFT was 148.43 days (95% CI [97.49, 199.38], *I*^*2*^ = 91.9%, *P* = 0.000), and EFI was 41.32 days/cm (95% CI [35.72, 46.91], *I*^*2*^ = 62.2%, *P* = 0.021), as presented in Figs. 3 and 4.

**Table 3:** Clinical outcomes of the included studies

Study	DS (cm)	EFT (days)	EFI (days/cm)	Bone union (%)	Complications (per patient)
Liu [8]	5.1 (4–6.5)	232.6 (182–276)	46.3 (40.9–61.8)	12/12 (100%)	2.08 (25/12)
Zhu [9]	3.54 (2.2–7.5)	195 (90–360)	51.6 (34.2–64.5)	19/19 (100%)	0.68 (13/19)
Ahmed [10]	2.79 (2.5–3.5)	103.3 (90–130)	37.0 (36.0–40.0)	12/12 (100%)	0.50 (6/12)
Mader [11]	2.64 (1.5–3.8)	70.7 (63–77)	27.6 (20.2–46.7)	10/10 (100%)	0.14 (1/7)
Zhang [12]	3.81 (2.2–7.5)	185.7 (90–300)	48.9 (34.2–60.0)	16/16 (100%)	2.19 (35/16)
Liu [13]	3.1 (1.8–4.6)	nr	42.5 (37.9–51.6)	21/21 (100%)	1.33 (28/21)
Smith [14]	4.2 (1.7–7.3)	210 (112–434)	nr	11/11 (100%)	1.1 (12/11)

*nr* Not reported

### Complications

Complications are summarized in Table 4. Liu et al. [9] reported the presence of axial deviation and soft tissue incarceration. Mader et al. [12] reported the occurrence of refracture. Zhang et al. [13] reported the occurrence of radial head dislocation. Liu et al. [14] reported the occurrence of pin loosening. For the complications reported in more than one study, including pin track infection, delayed union or nonunion, joint stiffness, and bone grafting, the pooled incidence was 51% (95% CI [37%, 65%], *I*^*2*^ = 52.5%, *P* = 0.061), 22% (95% CI [12%, 33%], *I*^*2*^ = 0, *P* = 0.738), 19% (95% CI [5%, 33%], *I*^*2*^ = 0, *P* = 0.468), and 29% (95% CI [16%, 42%], *I*^*2*^ = 0, *P* = 0.972), respectively. According to the aforementioned analysis, pin tract infection exhibited the highest incidence. The results of stratified meta-analyses about complications based on the average value of DS and EFT are presented in Tables 5 and 6.

**Table 4:** Meta-analysis of complications

Complications	Relevant studies (*n*)	Heterogeneity (*I*^2^,%; *P*)	ES (95% CI)	Range of incidence (%)
Pin track infection	6	*I*^2^ = 52.5; *P* = 0.061	0.51 (0.37, 0.65)	18.0–66.7
Axial deviation	1	–	–	8.3
Soft tissue incarceration	1	–	–	8.3
Delayed union or Nonunion	4	*I*^2^ = 0; *P* = 0.738	0.22 (0.12, 0.33)	18.8–36.0
Joint stiffness	2	*I*^2^ = 0; *P* = 0.468	0.19 (0.05, 0.33)	27.2–33.3
Refracture	1	–	–	14.3
Bone grafting	3	*I*^2^ = 0; *P* = 0.972	0.29 (0.16, 0.42)	27.3–31.3
Dislocation of the radial head	1	–	–	6.0
Pin loosening	1	–	–	28.6

**Table 5:** Stratified meta-analysis of complications based on DS

Complications	Relevant studies (*n*)	Heterogeneity (*I*^2^,%; *P*)	ES (95% CI)	Range of incidence (%)
Mean DS ≥ 3.60 cm				
Pin track infection	3	*I*^2^ = 79.7; *P* = 0.018	0.49 (0.17, 0.80)	18.0–66.7
Delayed union or Nonunion	3	*I*^2^ = 0; *P* = 0.601	0.24 (0.11, 0.38)	18.8–36.0
Bone grafting	2	*I*^2^ = 0; *P* = 0.823	0.30 (0.12, 0.47)	27.3–31.3
Mean DS < 3.60 cm				
Pin track infection	3	*I*^2^ = 0; *P* = 0.916	0.54 (0.40, 0.67)	50.0–57.1
Delayed union or Nonunion	1	–	0.19 (0.02, 0.36)	19
Bone grafting	1	–	0.29 (0.09, 0.48)	28.6

**Table 6:** Stratified meta-analysis of complications based on EFT

Complications	Relevant studies (*n*)	Heterogeneity (I^2^, %; *P*)	ES (95% CI)	Range of incidence (%)
Mean EFT ≥ 166.22 days				
Pin track infection	4	*I*^2^ = 70.1; *P* = 0.018	0.50 (0.28, 0.71)	18.0–66.7
Delayed union or Nonunion	3	*I*^2^ = 0; *P* = 0.601	0.24 (0.11, 0.38)	18.8–36.0
Bone grafting	2	*I*^2^ = 0; *P* = 0.823	0.30 (0.12, 0.47)	27.3–31.3
Mean EFT < 166.22 days				
Pin track infection	2	*I*^2^ = 0; *P* = 0.692	0.55 (0.38, 0.72)	50.0–57.1
Delayed union or Nonunion	–	–	–	–
Bone grafting	–	–	–	–

### Sensitivity analysis and publication bias

**Fig. 5:**
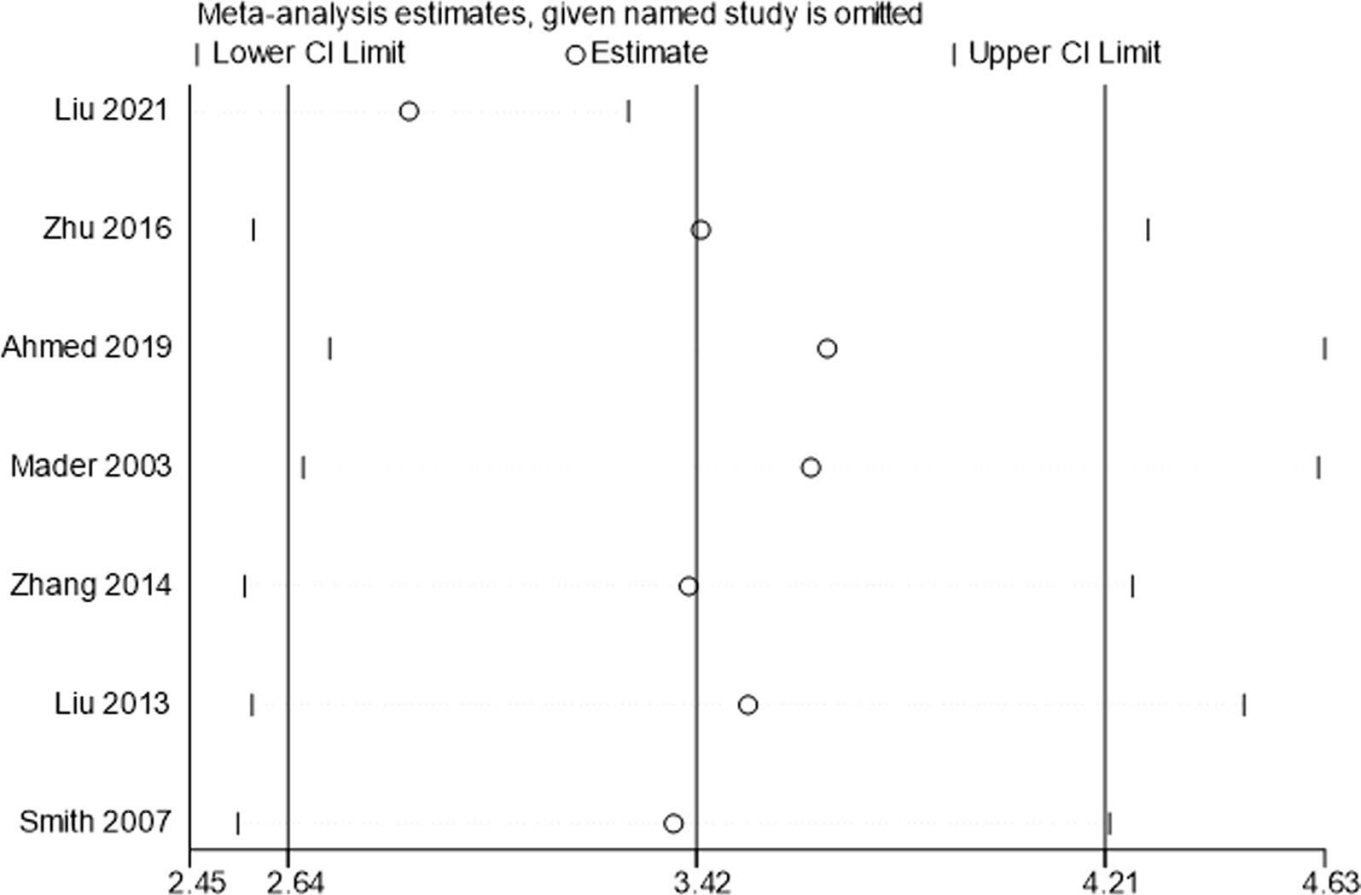
A sensitivity analysis for the summarized estimates of DS

**Fig. 6:**
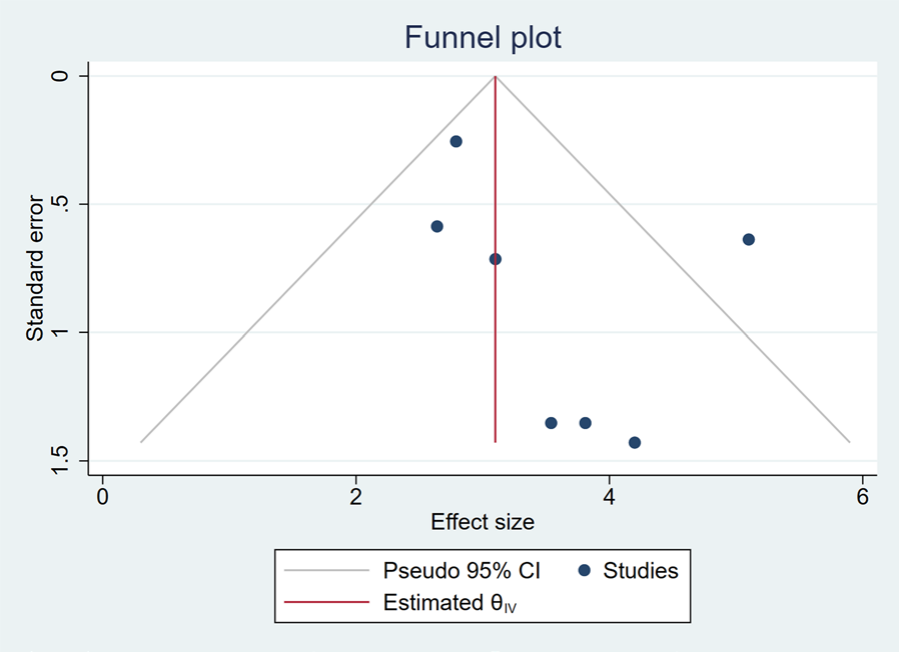
Funnel plots for publication bias of the overall pooled DS

A sensitivity analysis for the summarized estimates of DS with seven studies was conducted to ensure the stability of the results. After removing each study, the results did not change (Fig. 5). The funnel plots for effect size were used to visually assess the presence of publication bias (Fig. 6).

## Discussion

The present study represents the first systematic review of bone defects related to the radius and ulna that have been managed through the utilization of the Ilizarov technique. The compiled findings of the research indicated a high bone union rate of 100%, and the results of the meta-analysis appear to demonstrate the capability of the Ilizarov method in the treatment of bone defects of the radius and ulna.

Various factors may contribute to the occurrence of abnormalities in the forearm, leading to the relatively typical outcome of a disparity in length between the radius and the ulna [1–14]. The primary objectives of the treatment encompass the restoration of appropriate length and alignment of the bones in the forearm, the attainment of optimal functionality in forearm outcomes, and the successful achievement of the bone union. Undoubtedly, the management of such conditions poses a challenging task for surgeons given the intricate nature of the forearm joint mechanism, which comprises more than just two distinct bones. The maintenance of anatomical integrity is crucial for the optimal functioning and coordinated action of the body, particularly with regard to pronation and supination [17].

For forearm defects with known causes, there are a variety of accepted treatments, including corticocancellous bone graft, nonvascularized fibular graft, vascularized fibular graft, Masquelet technique, and Ilizarov methods [8–14, 18–22]. The Ilizarov technique is a minimally invasive and effective method that is preferred for the treatment of the aforementioned condition due to its ability to preserve the necessary biomechanical microenvironment required for healing [8–14]. Esser et al. [1] had previously used segmental bone transport to repair a posttraumatic lesion in a patient's forearm as early as 1996. The surgical intervention resulted in full osseous recuperation, enabling the patient to recommence his occupational duties.

In the current study, six studies have documented the rate of pin track infection, with a range of 18.0–66.7%. The pooled incidence of pin track infection was found to be 51% (95% CI [37%, 65%], *I*^2^ = 52.5%, *P* = 0.061) [8–10, 12–14]. One potential problem with the Ilizarov method is the total amount of time it takes for the external fixation, which in our study was 148.43 days (95% CI [97.49, 199.38], *I*^2^ = 91.9%, *P* = 0.000). This can lead to problems such as the occurrence of pin site infections. This issue has been recommended by multiple osteotomy sites within the same bone segment [23–25]. When compared to bifocal bone transport, the EFT can be successfully decreased, and good bone outcomes obtained with the use of multifocal bone transport with multilevel osteotomy, as indicated by Borzunov et al. [23]. Yushan et al. [24] compared trifocal to bifocal bone transport for the treatment of prolonged tibial bone defects and found that the former significantly reduced the time needed for repair and the difficulties that came along with it. Another comparative study demonstrated that when tetrafocal and pentafocal bone transport is used, it could shorten the distraction period, fasten regeneration, and reduce the associated complications [25].

Delayed union or nonunion at the docking site was a relatively common complication, with a range of 18.8–36.0%. The pooled incidence was found to be 22% (95% CI [12%, 33%], *I*^2^ = 0, *P* = 0.738) [8, 12–14]. Bone grafting for delayed union or nonunion at the docking site was reported in three studies, with a pooled incidence of 29% (95% CI [16%, 42%], *I*^2^ = 0, *P* = 0.972). A debate exists regarding the standard practice of performing bone grafting at the docking site. Although cyclic distraction and compression have been shown to be effective in treating delayed union, certain authors still recommend bone grafting at the docking site following the distraction phase [23, 26, 27]. According to Giotakis et al. [28], the necessity of bone grafting can be obviated by ensuring that the docking site clearance and preparation result in the creation of two coapted surfaces with a substantial surface area contact. Paley et al. [29] reported that all 25 patients with tibial nonunion and bone defects achieved bone union at the docking sites with only compression and no bone grafts.

Despite the comprehensive and systematic evaluation of the Ilizarov technique's efficacy in treating bone defects of the radius and ulna in this study, certain limitations remain. The sample size in the studies involved was small, and all seven studies were retrospective case series. The absence of detailed records related to bone and functional outcomes in the studies under consideration precludes the possibility of a comprehensive analysis of the various variables. This, in turn, may introduce a degree of bias in the conclusions that can be drawn from the data. Therefore, further research utilizing a prospective, large-scale, and multicenter clinical study is warranted.

## Conclusion

The study demonstrated that the utilization of the Ilizarov technique in patients with radius and ulna bone defects resulted in a noteworthy bone union rate of 100%. This finding suggests that the application of Ilizarov methods that rely on distraction osteogenesis may serve as a viable and efficacious alternative treatment approach for these types of conditions.

### Supplementary Information

**Additional file 1.** Search terms used for the individual databases.

## Data Availability

All data generated or analyzed during this study are included in this published article.
