# Supplementary material for: Ilizarov technique in the treatment of bone defects of the radius and ulna: a systematic review and meta-analysis
Source: J Orthop Surg Res. 2023 Aug 30;18:642. doi: 10.1186/s13018-023-04126-4 (PMC10469416; doi:10.1186/s13018-023-04126-4)
Supplement: Supplementary file 1 — Additional file 1. Search terms used for the individual databases. [file 13018_2023_4126_MOESM1_ESM.docx]

**Search terms used for the individual databases**

**PubMed**

****(("Ilizarov Technique"[MeSH Terms]) OR (Technique, Ilizarov) OR (Ilizarov Technic) OR (Technic, Ilizarov) OR (Ilizarov Method) OR (Method, Ilizarov)) OR (((Distraction Osteogenesis) OR (Distraction Osteogeneses) OR (Osteogeneses, Distraction)) OR ("Osteogenesis, Distraction"[Mesh])) AND ((bone loss) OR (bone defect)) AND (upper extremity)****

("Ilizarov Technique"[MeSH Terms] OR ("Ilizarov Technique"[MeSH Terms] OR ("ilizarov"[All Fields] AND "technique"[All Fields]) OR "Ilizarov Technique"[All Fields] OR ("technique"[All Fields] AND "ilizarov"[All Fields]) OR "technique ilizarov"[All Fields]) OR ("Ilizarov Technique"[MeSH Terms] OR ("ilizarov"[All Fields] AND "technique"[All Fields]) OR "Ilizarov Technique"[All Fields] OR ("ilizarov"[All Fields] AND "technic"[All Fields]) OR "ilizarov technic"[All Fields]) OR ("Ilizarov Technique"[MeSH Terms] OR ("ilizarov"[All Fields] AND "technique"[All Fields]) OR "Ilizarov Technique"[All Fields] OR ("technic"[All Fields] AND "ilizarov"[All Fields])) OR ("Ilizarov Technique"[MeSH Terms] OR ("ilizarov"[All Fields] AND "technique"[All Fields]) OR "Ilizarov Technique"[All Fields] OR ("ilizarov"[All Fields] AND "method"[All Fields]) OR "ilizarov method"[All Fields]) OR ("Ilizarov Technique"[MeSH Terms] OR ("ilizarov"[All Fields] AND "technique"[All Fields]) OR "Ilizarov Technique"[All Fields] OR ("method"[All Fields] AND "ilizarov"[All Fields])) OR ("osteogenesis, distraction"[MeSH Terms] OR ("osteogenesis"[All Fields] AND "distraction"[All Fields]) OR "distraction osteogenesis"[All Fields] OR ("distraction"[All Fields] AND "osteogenesis"[All Fields]) OR ("osteogenesis, distraction"[MeSH Terms] OR ("osteogenesis"[All Fields] AND "distraction"[All Fields]) OR "distraction osteogenesis"[All Fields] OR ("distraction"[All Fields] AND "osteogeneses"[All Fields])) OR ("osteogenesis, distraction"[MeSH Terms] OR ("osteogenesis"[All Fields] AND "distraction"[All Fields]) OR "distraction osteogenesis"[All Fields] OR ("osteogeneses"[All Fields] AND "distraction"[All Fields])) OR "osteogenesis, distraction"[MeSH Terms])) AND ("bone diseases, metabolic"[MeSH Terms] OR ("bone"[All Fields] AND "diseases"[All Fields] AND "metabolic"[All Fields]) OR "metabolic bone diseases"[All Fields] OR ("bone"[All Fields] AND "loss"[All Fields]) OR "bone loss"[All Fields] OR (("bone and bones"[MeSH Terms] OR ("bone"[All Fields] AND "bones"[All Fields]) OR "bone and bones"[All Fields] OR "bone"[All Fields]) AND ("abnormalities"[MeSH Subheading] OR "abnormalities"[All Fields] OR "defects"[All Fields] OR "defect"[All Fields] OR "defect s"[All Fields] OR "defected"[All Fields] OR "defective"[All Fields] OR "defectively"[All Fields] OR "defectives"[All Fields]))) AND ("upper extremity"[MeSH Terms] OR ("upper"[All Fields] AND "extremity"[All Fields]) OR "upper extremity"[All Fields])

**Cochrane Library**

((Ilizarov technique) OR (distraction osteogenesis)) AND ((bone loss) OR (bone defect)) AND (upper extremity)

**Embase**

(**'ilizarov technique'**/exp OR **'ilizarov technique'** OR ((**'ilizarov'**/exp OR **ilizarov**) AND (**'technique'**/exp OR **technique**)) OR **'distraction osteogenesis'**/exp OR **'distraction osteogenesis'** OR ((**'distraction'**/exp OR **distraction**) AND (**'osteogenesis'**/exp OR **osteogenesis**))) AND (**'bone loss'**/exp OR **'bone loss'** OR ((**'bone'**/exp OR **bone**) AND (**'loss'**/exp OR **loss**)) OR **'bone defect'**/exp OR **'bone defect'** OR ((**'bone'**/exp OR **bone**) AND **defect**)) AND (**'upper extremity'**/exp OR **'upper extremity'** OR (**upper** AND (**'extremity'**/exp OR **extremity**)))

**Ovid MEDLINE**

((Ilizarov technique or distraction osteogenesis) and (bone defect or bone loss) and upper extremity).ti,ab.

**Web of Science**

(ALL=(Ilizarov technique) OR ALL=(distraction osteogenesis)) AND (ALL=(bone defect) OR ALL=(bone loss)) AND ALL=(upper extremity)

**Scopus**

( TITLE-ABS-KEY ( "Ilizarov technique" OR "distraction osteogenesis" ) AND TITLE-ABS-KEY ( "bone loss" OR "bone defect" ) AND TITLE-ABS-KEY ( "upper extremity" ) )
